# Upper respiratory tract microbiome profiles in SARS-CoV-2 Delta and Omicron infected patients exhibit variant specific patterns and robust prediction of disease groups

**DOI:** 10.1128/spectrum.02368-23

**Published:** 2023-10-31

**Authors:** Shankha Nath, Mousumi Sarkar, Ankita Maddheshiya, Debjit De, Shouvik Paul, Souradeep Dey, Kuhu Pal, Suman Kr. Roy, Ayan Ghosh, Sharmila Sengupta, Suman Kalyan Paine, Nidhan K. Biswas, Analabha Basu, Souvik Mukherjee

**Affiliations:** 1 National Institute of Biomedical Genomics, Kalyani, West Bengal, India; 2 Department of Community Medicine, College of Medicine and JNM Hospital, Kalyani, West Bengal, India; 3 Department of Microbiology, College of Medicine and JNM Hospital, Kalyani, West Bengal, India; Karolinska Institutet, Huddinge, Stockholm, Sweden

**Keywords:** COVID-19, microbiome, Omicron, Delta, URT, next generation sequencing

## Abstract

The pandemic caused by the SARS-CoV-2 virus is the reason for many deaths globally. It is hypothesized that the upper respiratory tract (URT) microbiome shares similarities with lung microbiota and can modulate host immune responses to the virus. During the pandemic, several SARS-CoV-2 variants have emerged with different clinical outcomes and immune dysfunction, yet their association with changes in the URT microbiome has not been identified, which may provide an assessment of lung health in the presence of those variants with differential disease outcomes. In this study, we sequenced V3–V4 region of the 16S rRNA gene from the URT microbiome of healthy controls (HC), Delta- and Omicron-infected patients from Eastern India, which showed higher inter-individual diversity (β) and lower intra-individual diversity (α) in COVID-19 patients compared to healthy controls. Healthy control microbiome showed enrichment of commensals like *Streptococcus symci*, *Prevotella melaninogenica*, *Neisseria perflava*, *Veillonella tobetsuensis, Veillonella nakazawae, Haemophilus parainfluenzae, Fusobacterium pseudoperiodonticum*, and *Bifidobacterium longum* compared to COVID-19 samples. Ct value of patients significantly positively correlated with *Streptococcus symci* and *Streptococcus toyakuensis* indicating a possible inverse relation with viral load. We also observed few bacterial taxa like *Staphylococcus caprae, Pseudomonas aeruginosa*, *Vibrio tritonious*, and *Rothia mucilaginosa* are discriminating Omicron from Delta which is, in turn, enriched mostly with *Enterobacter mori*, *Acinetobacter baumannii,* and *Klebsiella pneumoniae*. Further investigation showed nine control-associated bacteria had higher to lower trends among three groups viz., Control > Omicron > Delta. These bacteria also contributed to the successful prediction of disease groups with high accuracy (90% ± 0.5%) by random forest analysis.

**IMPORTANCE:**

The role of the upper respiratory tract (URT) microbiome in predicting lung health has been documented in several studies. The dysbiosis in COVID patients has been associated with disease outcomes by modulating the host immune system. However, although it has been known that different SARS-CoV-2 variants manifest distinct transmissibility and mortality rates in human populations, their effect on the composition and diversity of the URT microbiome has not been studied to date. Unlike the older variant (Delta), the newer variant (Omicron) have become more transmissible with lesser mortality and the symptoms have also changed significantly. Hence, in the present study, we have investigated the change in the URT microbiome associated with Delta and Omicron variants and identified variant-specific signatures that will be useful in the assessment of lung health and can be utilized for nasal probiotic therapy in the future.

## INTRODUCTION

SARS-CoV-2 infection has emerged as a major public health concern in the 21st century. This novel coronavirus disease, known as COVID-19, was first reported from Wuhan, China, and since then has rapidly spread globally in almost every country (1). According to WHO, till 29 July 2022 approximately 572 million people have contracted COVID-19 and 6 million have succumbed to death globally (2). In India, a total of ~44 million people have been affected and ~0.5 million deaths have occurred due to COVID-19 to date (3). The major route of infection for COVID-19 is through the nasal cavity. At first, the virus accumulates in the nasopharynx and/or oropharynx and then aspirates into the lungs causing more serious infection (4). Hence, it is of immense importance to maintain nasal and oral health during COVID-19 infection to prevent severe outcomes (5). A healthy nasal and/or oral microbiome, collectively termed as the upper respiratory tract (URT) microbiome, is considered to be a protector of respiratory health and can provide resistance to invading pathogens (6). Alterations in the URT microbiome can potentially change the host immune response against viral and secondary bacterial infections (7, 8). Several 16S rRNA gene sequencing and a few RNA seq-based studies from Europe, Asia, and America on the URT microbiome of COVID-19 patients have been reported. Most of the studies have observed that specific genera like *Acinetobacter*, *Pseudomonas*, *Staphylococcus*, *Fusobacterium,* etc. are high in URT of patients (9 – 23).

Since the beginning of the COVID-19 pandemic, several prominent variants of SARS-CoV-2 have spread globally, including Alpha, Beta, Delta, and Omicron. Alpha and Beta both occurred in South Africa and the UK in late 2020, respectively (24). Delta was first identified in India in late 2020 and became the major cause of a disastrous second wave in March–June 2021 killing more than 0.4 million people in the country (25). In late 2021, Omicron and its sub-variants were identified in South Africa and since then it has been the major variant dominating globally (26). Delta resulted in a more severe form of the disease requiring oxygen and hospitalization compared to the newer variant Omicron, which showed relatively milder symptoms but with greater transmissibility (27). It is evident from previous studies that the URT microbiome significantly overlaps with the lung microbiome thereby considered as a good predictor of overall lung health (7). On the other hand, different variants of SARS-CoV-2 with varying symptoms and host immunological responses might have an impact on the URT microbiome further impacting lung health. The majority of the studies published to date have mostly characterized the URT microbiome in COVID-19 patients in comparison to healthy controls but the effect of specific SARS-CoV-2 variants on driving dysbiosis in the URT has not been identified before (18). In our current study, we aimed to investigate the changes in the URT microbiome composition and diversity associated with either the Delta or the Omicron SARS-CoV-2 variant in COVID-19 patients and compared the same with that of healthy controls. To the best of our knowledge, such extensive results on the variant-specific signatures of the URT microbiome have never been reported in COVID-19 patients globally.

## RESULTS

### Study participants and their characteristics

In total, 43 COVID-19 patients (24 Delta and 19 Omicron) and 19 HC were recruited in this study. All 19 Omicron and 5 of the delta samples were collected by the clinicians of COM&JNM Hospital in Kalyani from December 2021 to January 2022 and the rest of the 19 Delta samples were collected from ICMR-Regional Medical Research Centre, Bhubaneswar during the COVID second wave and published recently by members of our group (28). Healthy controls were collected from Kalyani during November–Dec 2021.

The average age of the patients and healthy controls was 35.95 Y ± 15.41 SD and 33.10 Y ± 12.26 SD, respectively. The average Ct value of the patients was 20.44 cycles ± 3.84 SD with 44% (19/43) having Ct < 20 (29). The Charlson comorbidity index was > 1 (min-max: 0–3) in 34.88% of patients (*n* = 15). Among the 43 patients, post-COVID complications were found as follows: (i) six patients developed diabetes, (ii) three developed chronic obstructive pulmonary disease, (iii) three reported post-COVID dementia, and (iv) two reported hypertension. Details of the clinical symptoms observed during COVID-19 infection for Delta and Omicron are given in Table 1.

**TABLE 1:** Clinical symptoms of Delta and Omicron variant-infected patients

Clinical symptoms	Delta-infected patients (*n* = 24)	Omicron-infected patients (*n* = 19)
Age	35 ± 18	37 ± 12
Gender (M:F)	13:11	13:5
Ct-value	22 ± 4	19 ± 3
Hypertension (%)	8.3 (2/24)	0.0 (0/19)
Diabetes (%)	20.8 (5/24)	5.3 (1/19)
COPD (%)	4.2 (1/24)	10.5 (2/19)
Charlson comorbidity index ≥ 1 (%)	37.5 (9/24)	26.3 (5/19)
Fever (%)	87.5 (21/24)	100 (19/19)
Cough (%)	91.7 (22/24)	78.9 (15/19)
Low SPO_2_ (%)	29.2 (7/24)	0.0 (0/19)
External O_2_ supplement (%)	29.2 (7/24)	0.0 (0/19)

### Lower microbial diversity was observed in patients compared to healthy controls

**Fig 1:**
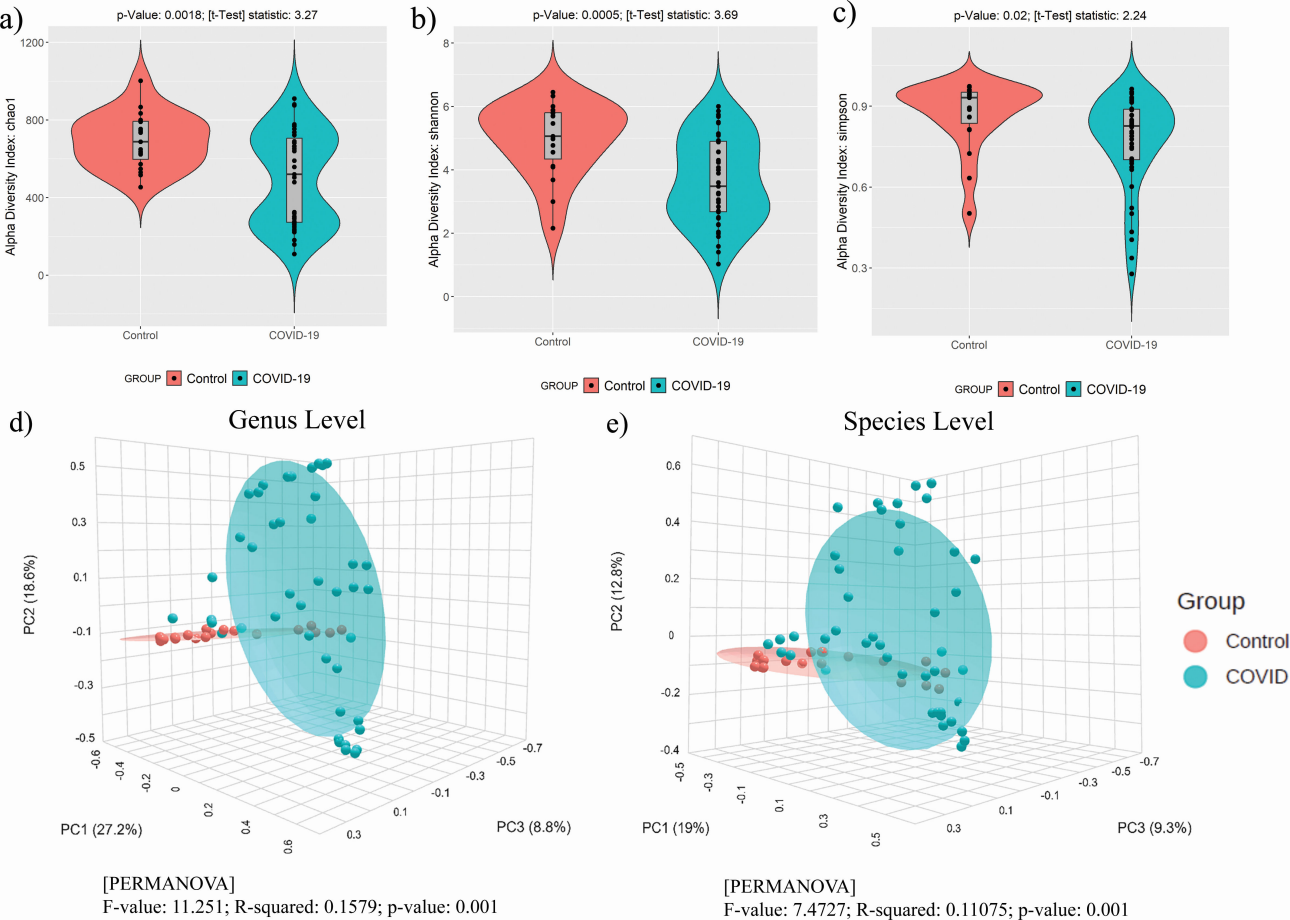
Difference in α- and β-diversity estimates between COVID-19 patients and healthy controls. (**a–c**) Violin plot showing groupwise (COVID-19, *n* = 43 and control, *n* = 19) difference of α-diversity estimates (Chao1, Shannon, and Simpson) (*P*-value < 0.05). (**d and **e) PCoA plot based on Bray-Curtis dissimilarity index of the microbial communities of each group (COVID-19 and control) at (d) genus and (e) species levels, respectively.

The total number of paired-end reads generated from all the 63 biospecimens (COVID-19: 43, healthy control: 19, and negative control: 1) sequenced was ~84.20 M (average ~1.33 ± 0.4 M). After initial QA/QC and adjustment for the negative control (supplementary information; Table S1), 44.24 M quality filtered, non-chimeric, singleton removed paired-end reads were obtained (maximum: 1.28 M, minimum: 0.29 M, and mean: 0.70 M) for amplicon sequence variant (ASV) binning, rarefaction analysis, and taxonomic classifications. The total ASV count without singletons was 12,172. Plateaued rarefaction curves (Supplementary information; Fig. S1) of α-diversity indices (Shannon and observed feature) were observed at ~40,000 reads, which is sufficient for our data as the sample with the minimum number of reads is 293,962. Intra-individual variability (α-diversity) measured by the Shannon, Chao1, and Simpson indices showed significantly higher (*P* < 0.01) microbiome diversity in healthy controls compared to COVID-19 patients (Fig. 1a through c).

Principal coordinate analysis (PCoA) using the β-diversity (Bray-Curtis dissimilarity index) showed that COVID-19 patients and healthy controls formed separate clusters at both the genus and species levels (Fig. 1d and e). We have used PCoA points for statistically comparing the PC1, PC2, and PC3 coordinate values between COVID patients and controls at both species and genus levels separately. We observed COVID-19 patients have significantly higher PC1 and PC2 coordinate values (Wilcoxon *P*-value < 0.05) compared to controls (Table S2). This shows a higher inter-individual variability across COVID-19 patients, whereas healthy control microbiome composition is mostly similar across individuals at both the genus and species levels.

### URT microbiome in COVID-19 patients are depleted for control-associated bacteria

A total of 34 phyla, 924 genera, and 1,429 species were observed. Among them, those taxa with mean relative abundance >1% and present in at least 50% of individuals in either COVID-19 patients or healthy controls were termed as the “core taxa.” The “core taxa” consists of 7 phyla, 34 genera, and 42 species (Table 2). These core phyla, genera, and species contributed ~99%, ~85%, and ~70% of the total relative abundance, respectively.

**TABLE 2:** Significantly abundant core genera and their respective mean relative abundance in COVID-19 infected patients, healthy controls, Delta, and Omicron patient groups

Phyla	Core genera under phyla	Healthy control (*n* = 19)	COVID-19 (*n* = 43)	LDA *P* value Kruskal–Wallis COVID vs control	Delta (*n* = 24)	Omicron (*n* = 19)	LDA *P* value Kruskal–Wallis Delta vs Omicron
		Mean relative abundance (%)	Mean relative abundance (%)		Mean relative abundance (%)	Mean relative abundance (%)	
Actinobacteriota	*Actinomyces*	0.462	0.562	1.27E-02	0.060	1.196	1.03E-06
	*Bifidobacterium*	3.252	0.586	2.26E-05	0.313	0.930	2.24E-03
	*Corynebacterium*	1.756	0.114	5.59E-05	0.012	0.242	3.30E-05
	*Rothia*	0.648	1.738	1.25E-02	0.033	3.891	3.92E-07
Bacteroidota	*Alloprevotella*	2.259	0.357	9.35E-08	0.023	0.778	2.53E-05
	*Capnocytophaga*	0.608	0.486	1.30E-04	0.047	1.041	6.39E-07
	*Chryseobacterium*	0.001	2.140	1.00E-06	3.823	0.013	2.81E-04
	*Porphyromonas*	4.220	0.799	–^*a*^	0.021	1.780	2.60E-06
	*Prevotella*	12.130	2.330	5.56E-06	0.392	4.778	3.98E-05
Campilobacterota	*Campylobacter*	1.100	0.236	2.00E-06	0.083	0.429	8.42E-05
Firmicutes	*Gemella*	1.159	0.204	2.98E-07	0.005	0.455	1.67E-07
	*Staphylococcus*	0.352	1.463	3.01E-05	0.011	3.297	3.83E-04
	*Streptococcus*	23.948	4.373	2.11E-05	1.796	7.629	4.42E-05
	*Veillonella*	11.438	3.361	6.12E-05	0.248	7.294	2.99E-06
Fusobacteriota	*Fusobacterium*	3.816	0.623	9.19E-07	0.088	1.298	1.08E-06
	*Leptotrichia*	2.476	0.809	4.63E-05	0.056	1.759	7.29E-07
Proteobacteria	*Acinetobacter*	0.049	6.002	3.95E-09	10.076	0.857	–
	*Actinobacillus*	6.332	0.025	4.82E-10	0.007	0.047	3.70E-02
	*Allorhizobium-Neorhizobium-Pararhizobium-Rhizobium*	0.002	1.778	1.56E-05	1.217	2.486	8.23E-05
	*Citrobacter*	0.001	1.607	1.19E-02	2.877	0.002	2.10E-03
	*Enterobacter*	0.025	15.651	–	28.036	0.007	1.76E-05
	*Haemophilus*	4.160	1.496	1.25E-06	1.510	1.478	5.64E-04
	*Halomonas*	0.000	1.271	3.73E-04	0.541	2.194	1.73E-05
	*Klebsiella*	0.138	7.675	–	13.747	0.006	8.48E-06
	*Moraxella*	2.886	0.002	8.46E-11	0.002	0.003	–
	*Neisseria*	6.868	1.074	8.15E-06	0.132	2.263	3.67E-06
	*Ochrobactrum*	0.000	0.864	4.38E-05	0.016	1.934	3.76E-07
	*Pelagibacterium*	0.000	2.324	1.18E-04	1.265	3.661	3.55E-04
	*Pseudoalteromonas*	0.000	1.182	7.26E-05	0.005	2.669	4.08E-07
	*Pseudomonas*	0.065	18.984	1.49E-05	14.400	24.774	2.95E-02
	*Rheinheimera*	0.004	1.264	–	0.370	2.395	1.22E-05
	*Serratia*	0.000	1.238	1.60E-09	2.209	0.012	1.50E-02
	*Stenotrophomonas*	0.001	2.094	1.80E-09	3.696	0.071	1.83E-04
	*Vibrio*	0.002	1.872	3.87E-02	0.002	4.235	1.35E-08

^*a*^ –, not significant.

**Fig 2:**
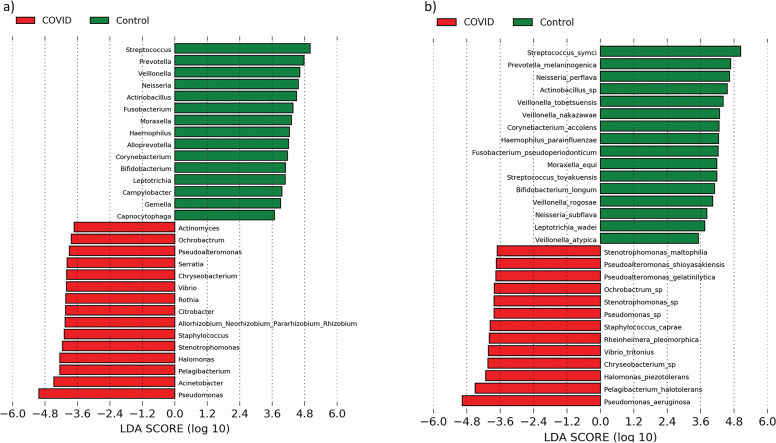
LEfSe analysis of core genus and species of two groups, i.e., COVID-19 patients and healthy controls. LEfSe analysis identified the most differentially abundant (a) genus and (b) species using LDA score > 3 and *P*-value < 0.05. “Green” color denotes taxa discriminating for the healthy control group (*n* = 19) and “red” color denotes taxa discriminating for the COVID-19 patients’ group (*n* = 43).

Linear discriminant analysis (LDA) combined with effect size (LEfSe) analysis revealed key bacterial taxa at phyla, genera, and species levels, which enabled discrimination (FDR-P_Kruskal–Wallis_<0.05 and LDA Threshold > 3) between COVID-19 patients and healthy controls. This analysis showed phylum Proteobacteria (COVID-19 positive: 73.57%, HC: 22.24%) is significantly predominant in COVID-19 patients, whereas healthy controls are predominated by three phyla [Firmicutes (COVID-19 positive: 12.03%, HC: 42,67%), Bacteroidota (COVID-19 positive: 9.11%, HC: 19.72%), and Fusobacteriota (COVID-19 positive: 1.43%, HC: 6.30%)]. The top five genera segregating the COVID-19 patients from the healthy controls are *Pseudomonas* (COVID-19 positive: 18.98%, HC: 0.07%)*, Acinetobacter* (COVID-19 positive: 6.00%, HC: 0.05%)*, Pelagibacterium* (COVID-19 positive: 2.32%, HC: <0.01%), *Halomonas* (COVID-19 positive: 1.27%, HC: <0.01%), and *Stenotrophomonas* (COVID-19 positive: 2.09%, HC: 0.01%) (Fig. 2a; Table 2). On the other hand, top five genera that predominated in healthy controls are *Streptococcus* (COVID-19 positive: 4.37%, HC: 23.94%)*, Neisseria* (COVID-19 positive: 1.07%, HC: 6.86%)*, Prevotella* (COVID-19 positive: 2.33%, HC: 12.13%), *Actinobacillus* (COVID-19 positive: 0.02%, HC: 6.33%)*,* and *Veillonella* (COVID-19 positive: 3.36%, HC: 11.43%) (Fig. 2a; Table 2).

At the species level, we observed that URT of COVID-19 patients is predominated by *Pseudomonas aeruginosa* (COVID-19 positive: 12.36%, HC: 0.02%), *Pelagibacterium halotolerans* (COVID-19 positive: 2.18%, HC: <0.01%), *Halomonas piezotolerans* (COVID-19 positive: 0.79%, HC: <0.01%), *Vibirio tritonious* (COVID-19 positive: 1.85%, HC: <0.01%), whereas healthy control URT is predominated by *Streptococcus symci* (COVID-19 positive: 19.66%, HC: 2.04%)*, Prevotella melaninogenica* (COVID-19 positive: 1.01%, HC: 7.28%), *Neisseria perflava* (COVID-19 positive: 0.75%, HC: 5.05%)*, Veillonella tobetsuensis* (COVID-19 positive: 0.74%, HC: 4.00%)*, Veillonella nakazawae* (COVID-19 positive: 1.22%, HC: 4.33%)*, Haemophilus parainfluenzae* (COVID-19 positive: 0.64%, HC: 2.82%)*, Fusobacterium pseudoperiodonticum* (COVID-19 positive: 0.53%, HC: 2.54%), and *Bifidobacterium longum* (COVID-19 positive: 0.44%, HC: 2.75%) (Fig. 2b; Table 2). They are mostly reported to be found in the oral and nasal cavities of healthy control individuals in previous studies (30 – 37), which support our observation that they are depleted in COVID-19 patients and abundant in the control individuals.

### Ct value is significantly positively correlated with *Streptococcus*

COVID-19 viral load as inferred by the Ct value was correlated with the core species to gain information on the relationship between viral load and URT microbiome composition. It was observed that *Streptococcus symci* and *Streptococcus toyakuensis* (Supplementary information; Fig. S2) are significantly positively correlated with Ct value (rho ≥ 0.3, *P* < 0.05), whereas oral non-commensal *Pseudomonas aeruginosa* (38) is significantly negatively correlated (rho ≤−0.4, *P* < 0.05) with Ct value (Supplementary information; Fig. S2). *Streptococcus* sp. is commonly found in the nasal cavity (30), and *Pseudomonas aeruginosa* is an oral pathogen (38, 39). This indicates that control-associated bacteria in URT are associated with higher Ct value and lower viral load whereas pathogenic *P. aeruginosa* is associated with an increase in viral load.

### URT microbiome diversity is lesser in Delta-infected patients compared to Omicron and healthy controls

**Fig 3:**
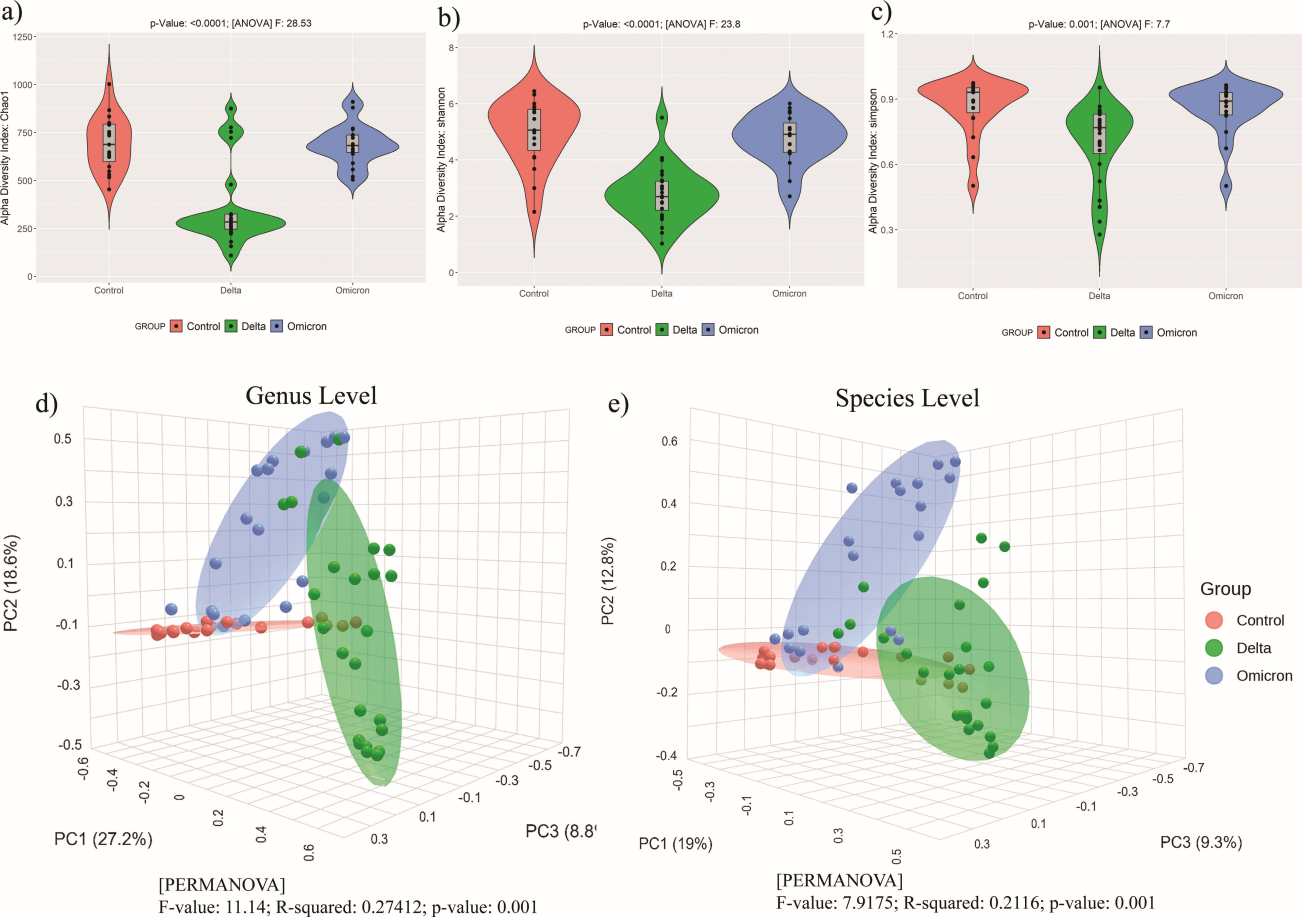
Difference in α- and β-diversity estimates among Delta-, Omicron-infected patients, and healthy controls. (a–c) Violin plot showing groupwise (Delta, Omicron, and healthy controls) difference of α-diversity estimates (Chao1, Shannon, and Simpson) (*P*-value <0.05). (**d and **e) PCoA plot based on Bray-Curtis dissimilarity index of the microbial communities of each group (Delta, Omicron, and healthy controls) at (d) genus and (e) species levels, respectively.

Estimates of intra-individual α-diversity indices show that Delta-infected patients have significantly lesser URT microbiome diversity compared to healthy controls and Omicron-infected patients (Fig. 3a through c). Whereas healthy control individuals showed significantly higher intra-individual diversity in URT microbiome composition compared to the two SARS-CoV-2 variant infected groups. This hints at a possible dysbiosis with the predominance of few bacteria in Delta-infected patients compared to healthy controls and Omicron-infected patients.

Inter-individual β-diversity index (Bray-Curtis) showed healthy controls, Delta-, and Omicron-infected patients clustering separately (Fig. 3d and e) at both genus and species levels. Interestingly, healthy controls clustered very closely indicating similar microbiome composition among them.

### Distinct URT microbiome composition in Delta- and Omicron-infected patients

**Fig 4:**
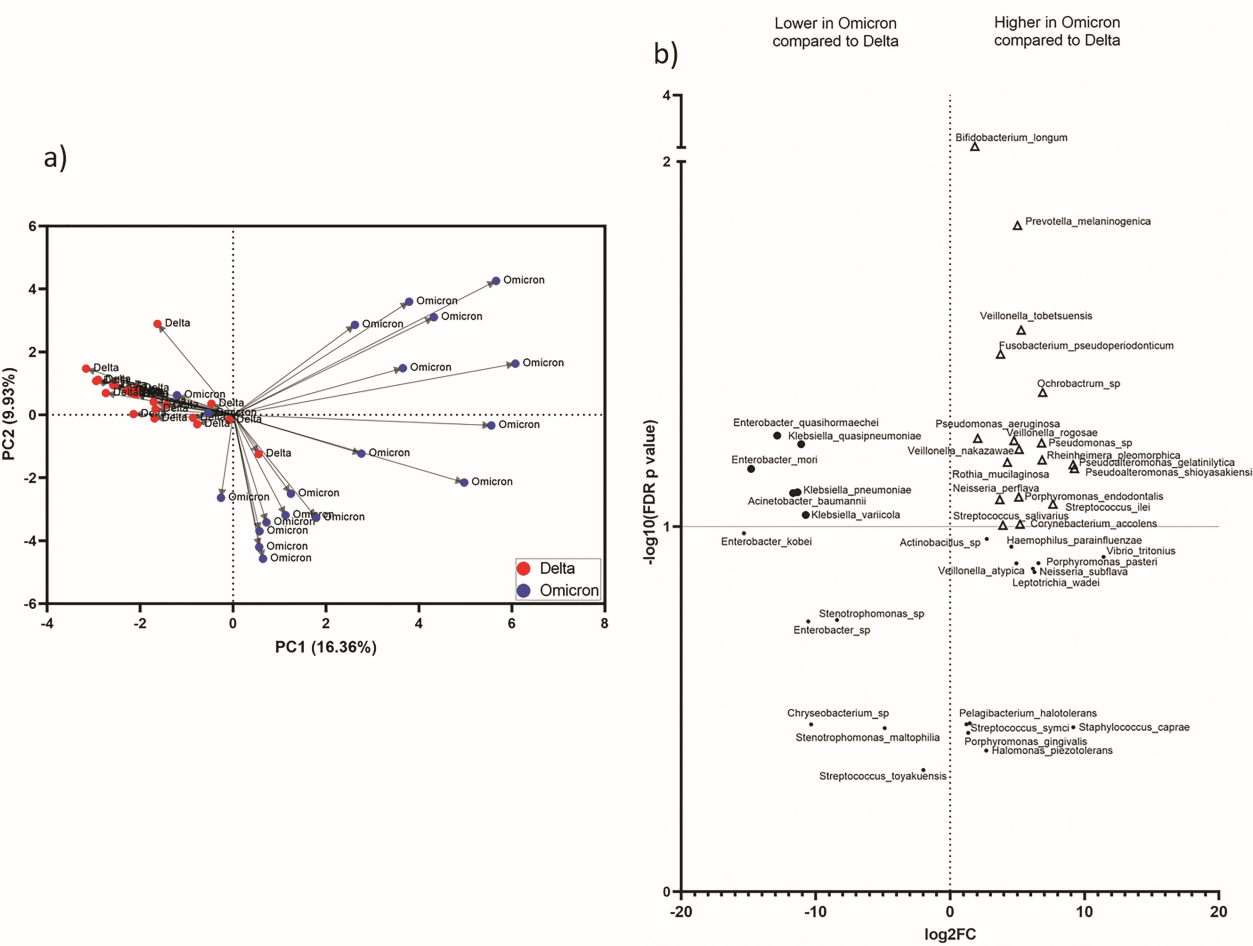
Taxonomic difference between Delta- and Omicron-infected patients. (a) PCA using core species level taxonomic data of Delta and Omicron variants infected patients’ samples. (b) Volcano plot to illustrate the significant association of microbial species with Omicron. The *X*-axis shows the estimate of the log of fold change (FC) of the mean relative abundance, and the *Y*-axis shows the −logarithm of the FDR *P*-value to the base 10. The species positively associated with the Omicron group are on the right side (“∆”) and those negatively associated are on the left side (“•”).

The URT microbiome of Delta-infected patients (n = 24) were compared to the Omicron-infected patients (*n* = 19) for identifying variant-specific signatures of the URT microbiome. Principal component analysis (PCA) on the core genera from both these data showed prominent clusters indicating distinct microbiome composition in both groups (Fig. 4a).

**Fig 5 F5:**
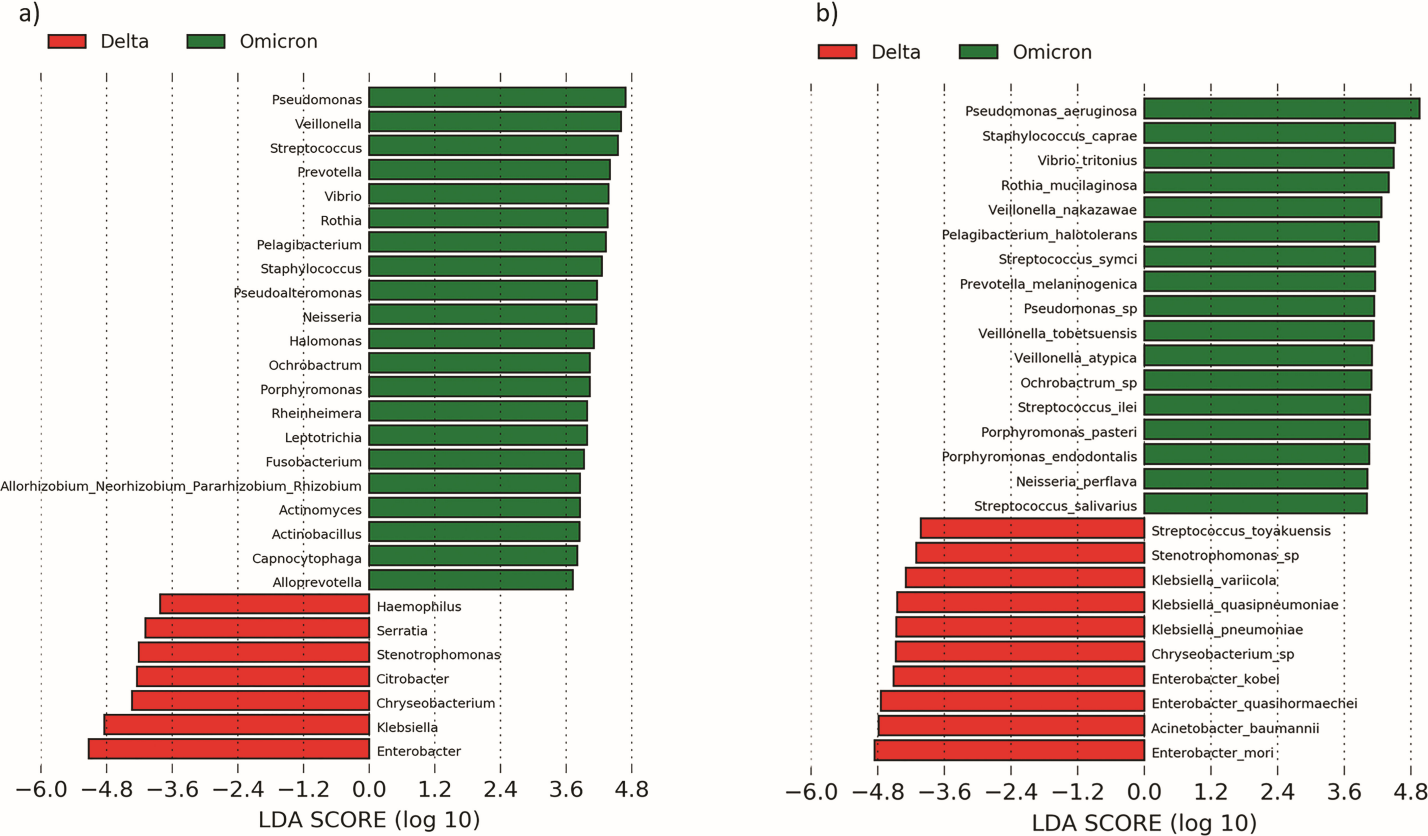
LEfSe analysis of core genus and species of two groups, i.e., Delta- and Omicron variant-infected patients. LEfSe analysis identified the most differentially abundant (a) genus and (b) species using LDA score > 3 and *P*-value < 0.05 are shown. “Green” color denotes taxa discriminating for Omircon-infected patients (*n* = 19), and “red” color denotes taxa discriminating for Delta-infected patients (*n* = 24).

To identify specific microbial taxa differentiating Delta and Omicron URT microbiome profiles, both multivariate (linear discriminant) and univariate (*t*-test) analyses were performed. Eleven species are found to be predominating (−log_10_P_FDR_ > 1 for univariate analysis and LDA threshold > 3, FDR-P_Kruskal–Wallis_ < 0.05 for multivariate analysis) in Omicron samples compared to Delta by both the analyses (Fig. 4b and 5 [Fig F5]). Among these species that are significantly discriminating Omicron samples from Delta, the top five are *Pseudomonas aeruginosa* (Omicron: 21.45%, Delta: 5.17%), *Rothia mucilaginosa* (Omicron: 3.74%, Delta: 0.03%), *Veillonella nakazawae* (Omicron: 2.68%, Delta: 0.07%), *Prevotella melaninogenica* (Omicron: 2.20%, Delta: 0.68%), and *Neisseria perflava* (Omicron: 1.56%, Delta: 0.12%) (Table 3).

**TABLE 3:** Significantly abundant core species and their respective mean relative abundance in COVID-19-infected patients, healthy controls, and Delta and Omicron patient groups

Phyla	Core species under phyla	Healthy control (*n* = 19)	COVID-19 (*n* = 43)	LDA *P*-value Kruskal–Wallis COVID vs control	Delta (*n* = 24)	Omicron (*n* = 19)	LDA *P*-value Kruskal–Wallis Delta vs Omicron
		Mean relative abundance (%)	Mean relative abundance (%)		Mean relative abundance (%)	Mean relative abundance (%)	
Actinobacteriota	*Bifidobacterium longum*	2.746	0.437	2.27E-04	0.203	0.731	5.30E-03
	*Corynebacterium accolens*	1.407	0.025	9.59E-06	0.003	0.053	4.96E-03
	*Rothia mucilaginosa*	0.598	1.672	–^*a*^	0.033	3.743	4.26E-06
Bacteroidota	*Chryseobacterium* sp.	0.001	2.096	4.77E-06	3.752	0.003	3.08E-04
	*Porphyromonas endodontalis*	1.016	0.019	–	0.001	0.041	8.05E-06
	*Porphyromonas gingivalis*	1.326	0.001	–	0.001	0.002	–
	*Porphyromonas pasteri*	1.801	0.736	–	0.017	1.644	4.67E-06
	*Prevotella melaninogenica*	7.288	1.012	3.41E-05	0.068	2.205	2.51E-06
Firmicutes	*Staphylococcus caprae*	0.295	0.999	1.10E-05	0.004	2.255	7.88E-03
	*Streptococcus ilei*	0.215	0.623	–	0.007	1.402	7.50E-06
	*Streptococcus salivarius*	0.434	0.706	–	0.042	1.546	6.17E-04
	*Streptococcus symci*	19.663	2.042	6.19E-07	1.287	2.995	2.22E-04
	*Streptococcus toyakuensis*	2.119	0.124	1.48E-06	0.185	0.047	5.01E-03
	*Veillonella atypica*	1.001	0.940	1.34E-04	0.067	2.042	4.15E-04
	*Veillonella nakazawae*	4.332	1.229	1.90E-04	0.077	2.685	2.94E-05
	*Veillonella rogosae*	1.847	0.405	7.50E-05	0.032	0.877	5.74E-05
	*Veillonella tobetsuensis*	4.007	0.742	6.55E-05	0.042	1.626	3.64E-05
Fusobacteriota	*Fusobacterium pseudoperiodonticum*	2.538	0.533	2.14E-04	0.082	1.103	4.81E-05
	*Leptotrichia wadei*	1.258	0.493	4.46E-05	0.014	1.097	3.05E-05
Proteobacteria	*Acinetobacter baumannii*	0.012	5.176	–	9.272	0.003	5.14E-06
	*Actinobacillus* sp.	6.310	0.024	5.89E-10	0.007	0.046	4.58E-02
	*Enterobacter kobei*	0.002	3.097	–	5.548	0.000	1.31E-06
	*Enterobacter mori*	0.005	6.757	–	12.106	0.000	1.73E-05
	*Enterobacter quasihormaechei*	0.014	4.291	–	7.688	0.001	3.07E-06
	*Enterobacter* sp.	0.001	0.586	–	1.050	0.001	1.50E-04
	*Haemophilus parainfluenzae*	2.818	0.648	3.09E-06	0.059	1.391	2.24E-03
	*Halomonas piezotolerans*	0.000	0.793	7.26E-05	0.231	1.504	3.10E-04
	*Klebsiella pneumoniae*	0.054	3.002	–	5.377	0.002	1.41E-06
	*Klebsiella quasipneumoniae*	0.058	2.539	–	4.547	0.002	8.65E-07
	*Klebsiella variicola*	0.025	1.886	–	3.377	0.002	2.49E-05
	*Moraxella equi*	2.876	0.001	5.13E-09	0.001	0.000	–
	*Neisseria perflava*	5.056	0.756	2.05E-05	0.120	1.559	4.32E-05
	*Neisseria subflava*	1.080	0.188	1.98E-03	0.006	0.418	2.60E-05
	*Ochrobactrum* sp.	0.000	0.864	4.38E-05	0.016	1.934	9.45E-07
	*Pelagibacterium halotolerans*	0.000	2.189	1.18E-04	1.232	3.397	5.24E-04
	*Pseudoalteromonas gelatinilytica*	0.000	0.619	1.18E-04	0.002	1.398	5.34E-06
	*Pseudoalteromonas shioyasakiensis*	0.000	0.563	7.26E-05	0.002	1.271	1.72E-06
	*Pseudomonas aeruginosa*	0.002	12.368	2.25E-08	5.171	21.458	2.63E-03
	*Pseudomonas* sp.	0.001	0.874	3.17E-06	0.017	1.956	1.80E-05
	*Rheinheimera pleomorphica*	0.000	1.096	7.26E-05	0.122	2.327	1.94E-05
	*Stenotrophomonas maltophilia*	0.000	0.857	2.61E-05	1.494	0.051	–
	*Stenotrophomonas* sp.	0.000	1.172	4.59E-06	2.095	0.006	9.99E-08
	*Vibrio tritonius*	0.000	1.857	7.26E-05	0.002	4.202	2.37E-07

^*a*^ –, not significant.

Non-commensal species like *Enterobacter mori* (Delta: 12.10%, Omicron: <0.01%), *Acinetobacter baumannii* (Delta: 9.27%, Omicron: <0.01%), *Enterobacter quasihormaechei* (Delta: 7.68%, Omicron: <0.01%), *Klebsiella pneumoniae* (Delta: 5.37%, Omicron: <0.01%), and *Klebsiella quasipneumoniae* (Delta: 4.54%, Omicron: <0.01%) (Table 3) are found to be significantly discriminating Delta samples from Omicron (Fig. 5). This shows a significant difference in the URT microbiome composition between Delta- and Omicron-infected patients.

Interestingly, among the top five species that are discriminating Omicron samples, *Prevotella melaninogenica, Neisseria perflava,* and *Veillonella nakazawae* are also previously found to be predominated in healthy control URT microbiome.

### Lesser dysbiosis in Omicron than Delta-infected patients with respect to healthy controls

**Fig 6:**
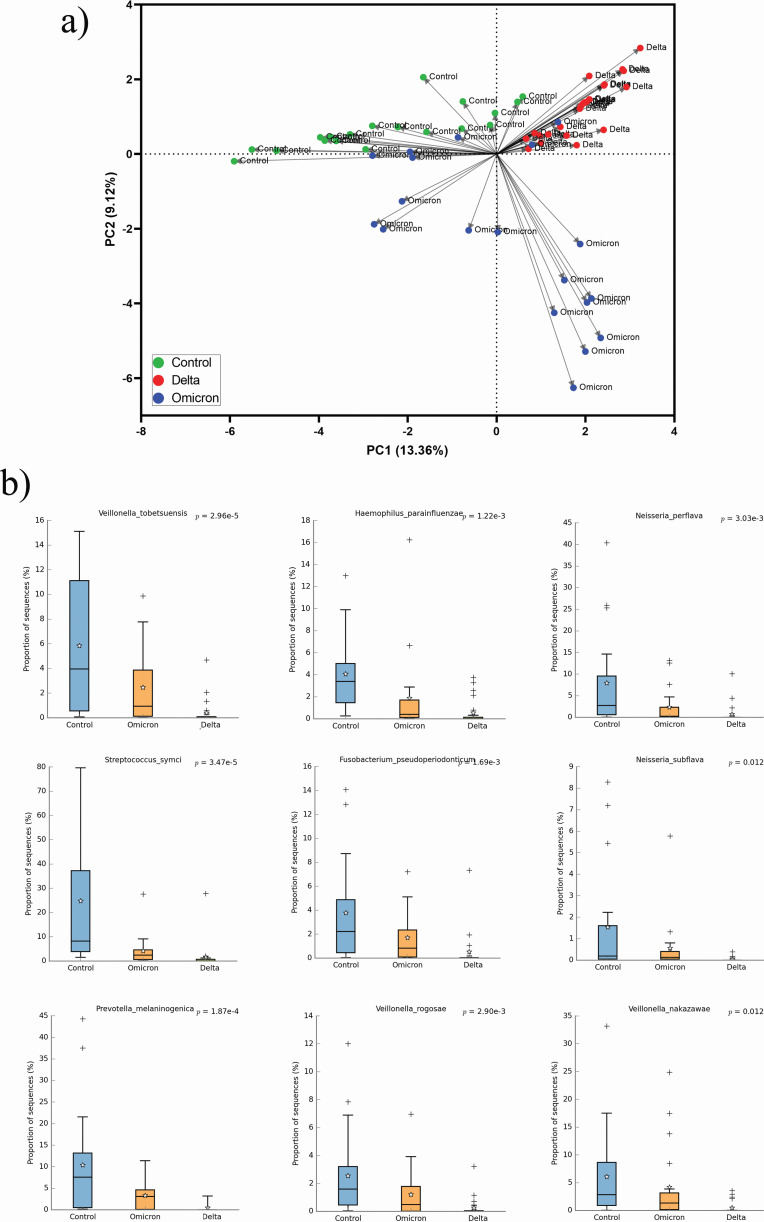
Taxonomic difference among healthy controls, Delta-, and Omicron-infected patients. (a) PCA using core species level taxonomic data of Delta samples, Omicron samples, and healthy controls. (b) Box and whisker plots showing the trend of significant decrease in abundance of nine key species from healthy controls to Omicron to Delta variant-infected samples (one-way ANOVA *P* value FDR < 0.05, *post hoc* test for linear trend *P* < 0.05).

To investigate the extent of dysbiosis in Omicron- and Delta-infected patients compared to healthy controls, we have performed PCA on the core species data for Delta (*n* = 24), Omicron (*n* = 19), and healthy control (*n* = 19) samples. The PCA plot (Fig. 6a) showed that Delta patients and healthy controls separately clustered along the PC1 (13.36%), while Omicron patients are mostly clustered along the PC2 (9.12%). Most importantly, the Delta samples revealed their clear distinctness from both the Omicron and healthy controls (Fig. 6a) by forming a prominent cluster in the PCA plot suggesting a clear dysbiosis present in them. We have compared the PCoA coordinate median values for PC1, PC2, and PC3 axes between (i) Delta-infected patients and healthy controls, and (ii) Omicron-infected patients and healthy controls. We observed that Delta patients have significantly higher PC1 and PC2 values (Wilcoxon *P*-value < 0.05) compared to controls. However, Omicron patients have significantly higher values only along the PC2 axis (Wilcoxon *P*-value < 0.05) and not along the PC1 axis. This shows that compared to healthy controls, the dysbiosis in Omicron samples is lesser as compared to Delta samples (Table S3).

To further investigate the key taxa that may be contributing to this difference in dysbiosis, we have performed one-way ANOVA and *post hoc* test for linear trend on the core species to determine the changes in relative abundance among the three groups, viz., healthy controls, Omicron-, and Delta-infected groups. This revealed nine key species *Streptococcus symci, Veillonella tobetsuensis*, *Veillonella rogosae*, *Veillonella nakazawae, Haemophilus parainfluenzae, Fusobacterium pseudoperiodonticum, Prevotella melaninogenica, Neisseria perflava,* and *Neisseria subflava* have trend of higher to lower abundance as control > Omicron > Delta (FDR-P_ANOVA_ <0.05, *post hoc* test for linear trend *P* < 0.05) (Fig. 6b; Fig. S3). These nine species showed a trend of regular decrease in abundance from healthy controls to Omicron- and Delta-infected patients. Notably, all these nine species were previously found to be significantly enriched in healthy controls when compared with the COVID-19 patients.

### Microbiome composition can effectively discriminate and predict Delta, Omicron, and healthy controls with 90% accuracy

**Fig 7:**
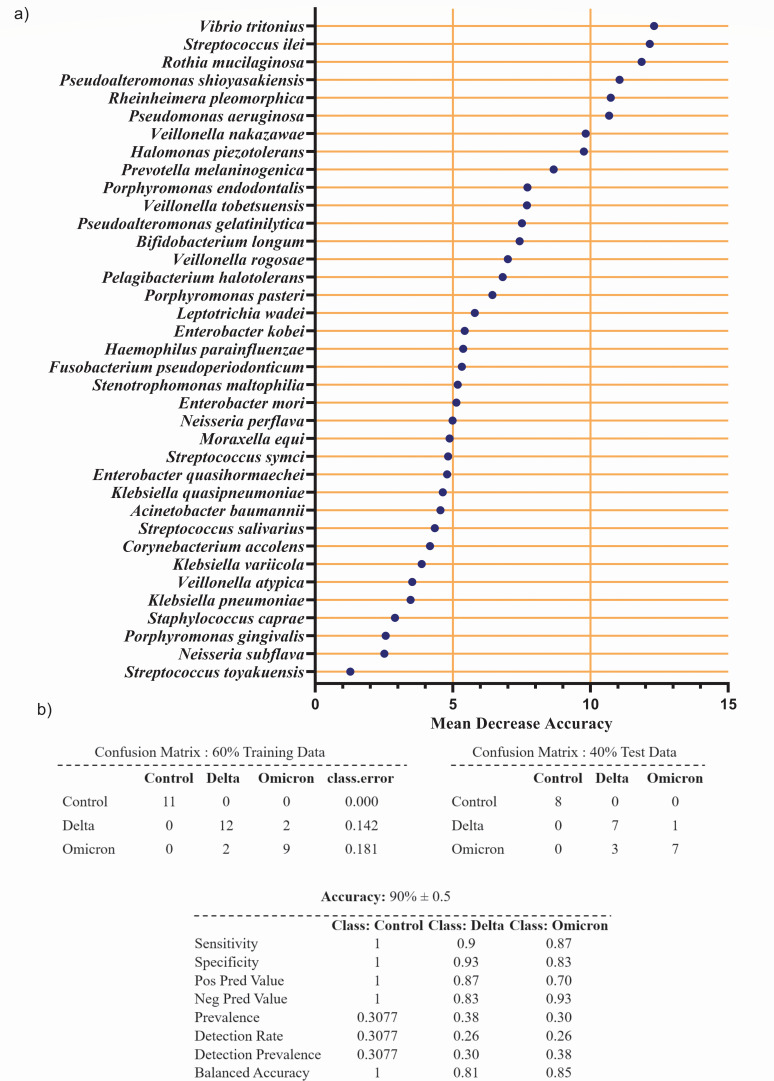
Random forest analysis using core species. (a) Variable importance plot based on mean decrease accuracy value from the random forest classifier between three groups (Delta, Omicron, and healthy control). (b) Confusion matrix showing prediction of three groups based on initial training data set (60% of the total data) and test data set (remaining 40% data).

To identify the robustness of discriminatory power of microbiome composition on the three groups (i) Delta (*n* = 24), (ii) Omicron (*n* = 19), and (iii) healthy controls (*n* = 19), random forest (RF) model-based prediction analysis was performed. The importance plot from the random forest classifier among the three groups showed these nine healthy control-associated key species *Streptococcus symci, Veillonella tobetsuensis*, *Veillonella rogosae*, *Veillonella nakazawae, Haemophilus parainfluenzae, Fusobacterium pseudoperiodonticum, Prevotella melaninogenica, Neisseria perflava,* and *Neisseria subflava* were contributing in successful prediction of the groups with ~90% (±0.5%) accuracy (Fig. 7a). This analysis suggested with a precise discriminatory power (class error; healthy control: 0%, Omicron: 18%, Delta: 14%; with 90% ± 0.5% accuracy) species level microbiome composition can distinguish healthy control, Delta, and Omicron patients (Fig. 7b).

## DISCUSSION

From the beginning of the pandemic till date, a number of studies have investigated the nasal or oral microbiome composition in COVID-19 patients and healthy controls (9 – 23). A majority of these studies have primarily investigated the association between microbiome composition and (i) disease severity or (ii) risk of developing SARS-CoV-2 infection (9, 15, 40 – 43). It has been well established that there is a significant overlap between URT and lung microbiome composition indicating a possible role of URT microbiome in overall lung health (6). Studies have shown that dysbiosis in the URT of COVID-19 patients certainly impacts lung health by influencing the host immunity (18). During the long course of the pandemic, newer variants of SARS-CoV-2 have emerged at different time points, with varying transmissibility and disease severity thus influencing lung health differentially, which can be deciphered from the composition and diversity of the URT microbiome of these patients. The newer variant Omicron is more transmissible with lesser mortality compared to other variants. The symptoms of Omicron infection and host immunological reactions are also milder compared to the older variant Delta (43). However, to the best of our knowledge, there is no study highlighting the difference in microbiome composition and diversity between patients infected by any of the two variants.

Since different SARS-CoV-2 variants are known to impact differently on the disease outcome, and the URT microbiome has been shown to modulate host response in COVID-19 patients, hence, in this current study, we hypothesized that SARS-CoV-2 Delta and Omicron-infected patients will exhibit variant-specific signatures in their URT microbiome that are also significantly different from that of the healthy controls. For this, we have initially compared the URT microbiome composition and diversity between COVID-19 patients and healthy controls and further characterized the dysbiosis associated with patients infected with Delta vs Omicron variants. URT microbiome diversity in COVID-19 patients is significantly lower compared to healthy controls supporting five previous studies (9, 10, 12, 21, 23) while refuting other three (11, 16, 20), which showed no changes in alpha diversity between COVID-19 patients and healthy controls. This can be attributed to the differences in geographical locations, ethnicity as well as sequencing approaches like metatranscriptomics, 16S, and shotgun metagenomic sequencing (9 – 23) undertaken by the respective studies.

To date, several methods have been applied based on the abundance or occupancy (i.e., detection across samples) to identify the core microbiome. A number of microbiome studies have used core criteria to be (i) taxa present in at least 50% of the individuals (44, 45) or (ii) taxa having at least 1% mean relative abundance (46) and performed further statistical analyses on them. However, several studies have also considered a combined criteria using microbial abundance and occurrence to be a robust method for core identification, which can explain more than 80% of the genera abundance (47, 48). Hence, we have considered both the criteria of abundance and occupancy for core microbiome identification as followed by our previous publication from the same lab (49). In this way, we have reduced the chance of including those rare taxa, which may have high abundance in only a few samples but absent in majority of the samples, thereby reducing the chance of getting false positives in the downstream analyses.

We have observed that *Streptococcus symci*, *Prevotella melaninognica*, *Neisseria perflava*, *Neisseria subflava, Bifidobacterium longum, Veillonella tobetsuensis, Veillonella nakazawae,* and *Corynebacterium accolens* can significantly discriminate the URT microbiome of healthy controls from COVID-19 patients. These bacteria were previously reported to be commonly found in the oral and nasal cavities of healthy humans (30 – 35, 50, 51). Previous reports have shown that *Neisseria subflava, Neisseria perflava*, and *Veillonella tobetsuensis* are the early colonizers (32, 33, 35) of the oral cavity and specifically *Veillonella tobetsuensis* coaggregates with *Streptococcus* sp. (35). Both *Veillonella tobetsuensis* and *Veillonella nakazawae* are known to metabolize lactate produced by other oral bacteria (52). This metabolic activity results in the production of less acidic byproducts, such as acetate, propionate, and carbon dioxide, which help buffer the oral environment. This acid-neutralizing effect contributes to the prevention of dental caries and enamel erosion by maintaining an optimal pH level in the oral cavity (53). *Neisseria subflava* and *Neisseria perflava* possess antimicrobial properties that can help protect the oral cavity from harmful pathogens by producing bacteriocins and antimicrobial peptides that inhibit the growth of competing bacteria, thereby limiting the colonization of potentially harmful microbes.

Ct value, or cycle threshold value, is a metric used in quantitative polymerase chain reaction assay. In the context of viral infections, including COVID-19, Ct values are used to estimate the viral load in a patient’s sample. A lower Ct value suggests a higher viral load, while a higher Ct value may indicate a lower viral load. We have found that *Streptococcus symci and Streptococcus toyakuensis* are significantly positively correlated with Ct value. On the contrary, *Pseudomonas aeruginosa* (11, 39), a known nasal and oral pathogen was found to be negatively correlated with Ct value. Similar observation was also reported for *Streptococcus* and *P. aeruginosa* by Rhoades et al. (11). A study by Gale et al. (54) showed a strong association between *Pseudomonas aeruginosa* and nasopharyngeal inflammation in RNA virus-infected immunocompromised patients with HIV. In these cases, *Pseudomonas aeruginosa* inversely correlated with CD4 cell count, which is crucial for immune regulation (54). Influenza virus infection has also been associated with increased susceptibility to *Pseudomonas aeruginosa* infection, contributing to lung damage through heightened MMP-9 expression (55, 56). *Pseudomonas aeruginosa* is also commonly known as a biofilm-forming opportunistic pathogen and a study from China showed a specific strain of *Pseudomonas aeruginosa* with unique epigenetic modifications could exhibit increased biofilm production thereby augmenting antibiotic resistance and facilitating *in vivo* colonization in COVID-19 patients (39). Conversely, many *Streptococcus* species possess adhesive molecules for their attachment to the epithelial layer of the upper respiratory tract (57). This hinders viral attachment to the host’s respiratory epithelial layer. They can also regulate oral pH by secreting both H_2_O_2_ and alkali molecules, thereby inhibiting foreign pathogen establishment (57). This indicates that an increase in *Streptococcus* sp. and pathogenic *P. aeruginosa* in the URT of patients is indicative of reduced and increased viral load, respectively.

We have compared our observation with other URT microbiome studies (number of studies = 7) (11, 12, 17, 19, 20, 58, 59) on COVID-19 patients and healthy controls who have only performed 16S rRNA gene sequencing (Supplementary information; Table S4). We have seen that in those studies *Acinetobacter*, *Pseudomonas,* and *Staphylococcus* are mostly found in the URT of COVID-19 patients (5/7; 70% of the studies) (Supplementary information; Table S4). Whereas *Prevotella, Neisseria*, and *Streptococcus* were commonly found in the URT of healthy controls (4/7; 57% of the studies). This is similar to our observation where we have found *Streptococcus*, *Prevotella,* and *Neisseria* to be discriminating control samples from COVID-19, whereas *Acinetobacter* and *Pseudomonas* were found to be discriminating COVID-19 samples from controls (Fig. 2a).

In our study, microbiome composition in the Delta- and Omicron-infected patients clearly showed distinct clusters along the PCA axes. Furthermore, both univariate and multivariate analyses showed that the URT of Omicron-infected patients is mostly predominated by healthy control-associated bacteria like *Streptococcus symci* (30)*, Prevotella melaninogenica* (20), *Neisseria perflava* (32, 33)*, Veillonella tobetsuensis* (35)*, Veillonella nakazawae* (34) along with non-commensal *Pseudomonas aeruginosa* (39), and potential pathogen *Staphylococcus caprae* (60), whereas those of Delta-infected patients showed a predominance of non-commensals like *Enterobacter mori* (61), *Acinetobacter baumannii* (62), *Enterobacter quasihormaechei* (63), *Klebsiella pneumoniae* (64), and *Klebsiella quasipneumoniae* (65).

Deeper investigation revealed nine key species *Streptococcus symci, Veillonella tobetsuensis*, *Veillonella rogosae*, *Veillonella nakazawae, Haemophilus parainfluenzae, Fusobacterium pseudoperiodonticum, Prevotella melaninogenica, Neisseria perflava, and Neisseria subflava,* which showed a trend of gradual decrease in abundance from healthy controls > Omicron > Delta-infected patients. All these species were previously found to be significantly high in healthy controls when compared to COVID-19 patients. Our analysis also indicates that compared to healthy controls, the extent of dysbiosis in the URT of Omicron-infected patients is lesser than in the Delta-infected patients with enrichment of few healthy URT-associated bacteria in the former compared to the latter.

One of the critical findings was to successfully predict the disease groups based on the microbiome composition with a very high accuracy (90% ± 0.5%). Random forest analysis has shown nine key species *Streptococcus symci, Veillonella tobetsuensis*, *Veillonella rogosae*, *Veillonella nakazawae, Haemophilus parainfluenzae, Fusobacterium pseudoperiodonticum, Prevotella melaninogenica, Neisseria perflava, and Neisseria subflava* along with other few noncommensal species, *Vibrio tritonis* and *Pseudomonas aeruginosa,* can effectively predict the Delta (specificity: 1), Omicron (specificity: 0.9), and healthy control (Specificity: 0.8) samples.

The objective of our prediction model is to identify the predictive power of the microbiome composition on different COVID-19 subtypes (Delta and Omicron) as well as healthy controls. Due to the lack of URT microbiome data specific to Delta and Omicron variant-infected patients in the existing literature and no available external data sets that could serve as relevant test data for our prediction model, we strategically partitioned our own data. We divided our data into a training set (60%) and a test set (40%) to assess the model’s performance as done in reference (66). We considered the test data as a new independent set as in reference (66). By training the random forest model on the training set and then evaluating it on the test set, we aimed to understand how well the discriminating microbial taxa for the Delta, Omicron, and healthy control based on a subset of our data can be used to predict test data. The division of data into random training and test sets helped to evaluate the model’s generalization capabilities. We also repeated the process 100 times thereby allowing us to access the robustness of the associations and gain insights into the potential diagnostic or biomarker value of these microbial features.

In a nutshell, our study provides evidence that the different SARS-CoV-2 variants emerging during the course of the pandemic have shaped the upper respiratory tract microbiome in a variant-specific manner, which might impact the overall lung health. This is an important addition to the growing scientific data on the COVID microbiome and will be useful for future studies focusing on studying host-microbiome interactions in COVID-19 patients. The variant-specific microbiome signatures can also lead to differences in host immunological responses in patients infected with either Delta or Omicron variants. Our study also highlights that the URT microbiome of patients infected by the newer variant Omicron are more similar to healthy controls than the older variant Delta-infected patients. This is an interesting observation hitherto unreported during the pandemic from any global population. This may have huge implications in studying the nature of the newer variants that might help in understanding the trajectory through which the dreaded pandemic is now moving towards an endemic condition on the planet.

Studies have shown that nasal probiotic spray can result in effective recovery in sinusitis and respiratory syncytial virus infection (67). Clinical studies have shown persistent *P. aeruginosa* colonization in gut and oral samples of COVID-19 patients (11, 39) supporting our observation as it is most predominant in Omicron-infected patients. On the other hand, oral *Bifidobacterium longum* supplementation is suggested for treating dental inflammation and influenza infection (37, 68). A functional study has also shown oral *Bifidobacterium longum* supplementation can prevent *P. aeruginosa*-driven infection in mice (69). We have observed a very low abundance (<0.5%) of *Bifidobacterium longum* in both Delta- and Omicron-infected patients. Hence, our findings can help in further development of nasal probiotic spray for COVID-19 patients taking into account different variants that are already prevailing in the population and also for the newer emerging variants.

Globally, there are seven URT microbiome studies on COVID samples done by 16S sequencing. Although our total sample size (*n* = 62) is greater than or comparable to four such studies (*n* = 19, *n* = 48, *n* = 59, and *n* = 71) (12, 17, 20, 59) but is lesser than the remaining three (*n* = 103, *n* = 134, and *n* = 141) (11, 19, 58) studies on COVID-19 URT microbiome (Table S4). Thus, our sample size might be a limitation of this study since it was logistically difficult during the pandemic period. However, despite these issues, our study represents a valuable initial investigation into the association between specific SARS-CoV-2 variants and the URT microbiome, and to the best of our knowledge, no other study has explored variant-specific microbiome profiles in COVID-19 patients.

## MATERIALS AND METHODS

### Study participants and sample collection

The Institutional Ethics Committee of College of Medicine and JNM Hospital, Kalyani, and National Institute of Biomedical Genomics, Kalyani have approved this study. Written informed consent was obtained from all patients and controls. URT swabs (19) from 5 Delta and 19 Omicron-infected patients were collected by clinicians of COM&JNM hospital in Kalyani during December 2021 and January 2022. The variant level information was obtained by viral RNA sequencing in NIBMG through INSACOG (70). Information on fever, cough, diarrhea, Ct value, and Charlson comorbidity index (71) was collected from all the patients by experienced clinicians. URT swabs of healthy volunteers were obtained from the same geographical region among those who have not reported positive for COVID-19 since the beginning of the pandemic. All the participants had not taken any antibiotics 1 month prior to sample collection. Another set of 19 delta samples have been collected from ICMR-Regional Medical Research Centre, Bhubaneswar during the COVID second wave and published recently by members of our group (28). Sequence data files from our previous study were jointly analyzed with the data generated in our current study for obtaining taxonomic profiles and diversity estimates using our analysis pipeline. All the participants did not have any oral infection or conditions like periodontitis or xerostomia.

### Microbiome DNA isolation and 16S rRNA gene sequencing

Microbiome DNA was isolated from the URT swabs collected from patients and healthy controls by using QIAamp BiOstic Bacteremia DNA Kit while maintaining proper Bio-Safety (BSL-II) guidelines followed in the COVID laboratory of COM&JNMH Hospital. Microbiome DNA was then transferred to NIBMG and stored at −20°C until further processing. Microbiome DNA was subjected to genomic characterization of both culturable and non-culturable microbial taxa by next-generation sequencing of variable regions (V3–V4) of 16S rRNA gene using paired-end (2 × 250 bp) sequencing chemistry on Illumina NovaSeq 6000 platform (supplementary information).

### Microbiome data analysis

Adapter sequences were removed during the demultiplexing of raw files obtained from the sequencer. After obtaining the demultiplexed individual sample files, FASTQC reports were generated to check the quality of the demultiplexed raw reads (http://www.bioinformatics.babraham.ac.uk/projects/fastqc) (72). All the downstream bioinformatics analysis was performed using QIIME2 (v2022.8) (73). The paired-end reads were imported in the QIIME2 environment using manifest file (73, 74). In the first step, denoising of the raw reads was performed using DADA2 (75) algorithm integrated in QIIME2 (73). The denoising step was performed by (i) removing the primers, (ii) not allowing any ambiguous base (*N* < 0), (iii) allowing read length > 250 bp, (iv) merging the paired-end reads with > 12 bp overlap, and (v) allowing error rate < 2. After the denoising step, ASV feature table (comprising sample IDs with quality filtered and non-chimeric read numbers) and representative sequences were generated. The representative sequences were then aligned with SILVA-v.138 (76) reference database using QIIME2 to obtain taxonomic classification based on VSEARCH (v 2.1.3) consensus taxonomy (73, 74, 77) classifier with default settings. Species identification was done by aligning the representative sequences obtained from ASVs by BLAST (78) and species were assigned only when the query fulfilled the BLAST identity > 99%. Rarefaction plots were generated with the actual number of reads for each sample to confirm if (i) the number of ASVs and (ii) the estimated α-diversity index (Shannon) were independent of the inter-individual variation of the total number of reads generated for each individual, i.e., reached a plateau even with a minimum number of reads. ASV feature table was further used for generating phylogenetic tree, α- and β-diversity estimation using QIIME2 diversity plugin. Negative control adjustment was done for BSL-II and DNA isolation kit by removing those ASVs from the cases and controls that are >1% in abundance in the negative control.

### Statistical analyses

The taxonomic data did not show any significant differences (*P*-value _PERMANOVA_ = 0.11) between the samples collected in COM&JNMH and ICMR-RMRC as evident by the PCoA plot generated using Bray-Curtis dissimilarity index, hence, the samples were pooled together for further statistical analysis (Supplementary information; Fig. S4).

α-diversity estimates were compared (i) between COVID-19 patients and healthy controls, and (ii) among Delta, Omicron, and healthy controls groups by Student’s *t* test and ANOVA, respectively, and violin plots were generated using R package ggplot2 (v3.4.2) (79). For comparing the beta diversity estimate, i.e., Bray-Curtis dissimilarity index for (i) and (ii) above, permutational MANOVA (PERMANOVA) was performed and PCoA plot was generated in R using Vegan (v2.6.4) and ggplot2 (v3.4.2) (79, 80).

Bacterial phyla, genera, and species with mean relative abundance >1% and present in at least 50% of individuals in either Omicron or Delta patients or healthy controls were termed the “core taxa” and these were carried forward for further analyses. Principal component analysis was performed on core taxa between study groups by R v4.1.1 using “PCAtools” (81) and “vegan” (80) packages. Correlation analysis between core taxa and Ct value for COVID-19 patients was performed using Spearman’s rank correlation test in R v4.1.1 using “dplyr” and “Hmisc” packages (82, 83). Regression analysis was performed using GraphPad Prism 8 (84) and subsequent plots were generated.

Multivariate linear discriminant analysis of effect size (85) was performed to obtain bacterial taxa significantly differentiating (i) COVID-19 patients from healthy controls, and (ii) Delta from Omicron groups. To identify those bacterial species that are significantly different between Delta and Omicron groups, we performed *t*-test for all the core species (*n* = 42) between the two groups and plotted the fold change in mean relative abundance for each taxon in *X*-axis and *P*-values in *Y*-axis represented in a volcano plot using GraphPad Prism 8 (84). To investigate whether any trend in the distribution of the relative abundance for members of the core species existed among the Control, Omicron, and Delta groups, one-way ANOVA and *post hoc* (Tukey-Kramer) test for linear trend was performed.

We hypothesized H_0_: there is no difference in the mean relative abundance of core species among the three groups (control, Omicron, and Delta) and H_A_: mean relative abundance of core species will be high in control followed by Omicron- and Delta-infected patients. RF analysis was performed to identify the discriminatory power of the members of the core species on the Delta, Omicron, and healthy controls. RF analysis was carried out with the help of R packages “caret” (86) (https://topepo.github.io/caret/) and “randomForest” (87) (https://cran.r-project.org/web/packages/randomForest/index.html) (details in supplementary information).

## Data Availability

16S data for 19 COVID Delta samples that are included in this study can be obtained from PRJNA850148. The rest of the raw sequence data for 16S can be found in the EBI-ENA portal using the accession PRJEB66856.

## SUPPLEMENTAL MATERIAL

Supplementary Information spectrum.02368-23-s0001.docx Contains Supplementary Methods, Figures and Tables.

ASM does not own the copyrights to Supplemental Material that may be linked to, or accessed through, an article. The authors have granted ASM a non-exclusive, world-wide license to publish the Supplemental Material files. Please contact the corresponding author directly for reuse.
