# Supplementary material for: Upper respiratory tract microbiome profiles in SARS-CoV-2 Delta and Omicron infected patients exhibit variant specific patterns and robust prediction of disease groups
Source: Microbiol Spectr. 2023 Oct 31;11(6):e02368-23. doi: 10.1128/spectrum.02368-23 (PMC10715160; doi:10.1128/spectrum.02368-23)
Supplement: Supplementary Information — Contains Supplementary Methods, Figures and Tables. [file spectrum.02368-23-s0001.docx]

**Supplementary Text**

**Sequencing Primers and Library Preparation Method**

The primers used for the amplification of the V3-V4 region was Illumina–F: 5’-CCTACGGGGNGGCWGCAG-3’ and Illumina–R: 5’-GACTACHVGGGTATCTAATCC-3’ with overhanging adapters attached to the 5’ end. Amplified PCR products were subsequently purified using Agencourt AMPure XP (Beckman Coulter) beads. Purified PCR products were quantified using Qubit^TM^ HS Assay Kit (Invitrogen) and quality control checks were performed using High Sensitivity D1000 Screen Tape (Agilent). Final index PCR was performed using the Nextera XT V2 Index Kit (Illumina) which uses a dual-indexing strategy involving 12 bp (i7) and 8 bp (i5) length indexes. After following a second round of PCR clean up, samples were finally pooled in equimolar concentrations (2nM) and the pooled library was further diluted. The final 4 picomolar (pM) denatured library was sequenced on NovaSeq 6000 using SP 500 kit with 25% PhiX DNA added as a spike-in control.

**Random Forest Analysis Details**

To perform Random Forest analysis the total dataset was randomly split into training and test sets in a ratio of 60%:40%. The training set along with 1000 decision trees, used to develop the prediction model and test set used to assess its performance. The whole analysis was also iterated 100 times and average accuracy and its standard error of the prediction model was measured. The mean decrease in accuracy of the RF model was chosen as a significant parameter for the purpose of better classifying Delta and Omicron infected patients and healthy controls. In order to demonstrate how well the classification models performed, the sensitivity and specificity was measured.

**Negative Control Adjustment Details**

We could select 10 ASVs which have >1% abundance in negative control. Amone these 5 were identified as uncultured bacteria. These 10 ASVs can explain 88.8% of the total abundance of the negative control. By removing these 10 ASVs from the total data we observed 0.011% (+0.034) reads were removed. Hence, we consider there is very less impact of this adjustment in the downstream analysis.

Table S1: ASV with >1% abundances in Negative control sample

| **Taxon** | **Negative Control  (ASV Abundance >1%)** |
| --- | --- |
| d__Bacteria; p__Bacteroidota; c__Bacteroidia; o__Flavobacteriales; f__Weeksellaceae; g__Chryseobacterium; s__Chryseobacterium_arthrosphaerae | 1.785 |
| d__Bacteria; p__Proteobacteria; c__Alphaproteobacteria; o__Caulobacterales; f__Caulobacteraceae; g__uncultured; s__uncultured_bacterium | 26.248 |
| d__Bacteria; p__Bdellovibrionota; c__Oligoflexia; o__0319-6G20; f__0319-6G20; g__0319-6G20; s__uncultured_bacterium | 4.828 |
| d__Bacteria; p__Proteobacteria; c__Alphaproteobacteria; o__Caulobacterales; f__Caulobacteraceae; g__uncultured; s__uncultured_bacterium | 16.000 |
| d__Bacteria; p__Desulfobacterota; c__Desulfovibrionia; o__Desulfovibrionales; f__Desulfovibrionaceae; g__Desulfovibrio; s__ | 2.396 |
| d__Bacteria; p__Proteobacteria; c__Alphaproteobacteria; o__Caulobacterales; f__Caulobacteraceae; g__uncultured; s__ | 26.809 |
| d__Bacteria; p__Proteobacteria; c__Alphaproteobacteria; o__Caulobacterales; f__Caulobacteraceae; g__uncultured; s__uncultured_bacterium | 2.528 |
| d__Bacteria; p__Proteobacteria; c__Alphaproteobacteria; o__Rhizobiales; f__Xanthobacteraceae; g__Bradyrhizobium; s__Bradyrhizobium_australafricanum | 2.924 |
| d__Bacteria; p__Bacteroidota; c__Bacteroidia; o__Chitinophagales; f__Chitinophagaceae; g__Sediminibacterium; s__ | 4.107 |
| d__Bacteria; p__Actinobacteriota; c__Actinobacteria; o__Micrococcales; f__Micrococcaceae; g__Micrococcus; s__Micrococcus_luteus | 1.187 |
| **Total Sum** | **88.812** |

Table S2: PCoA co-ordinate values for PC1, PC2 and PC3 axes among COVID-19 infected patients and Healthy controls

| **At Species level** | **PC1** | **PC2** | **PC3** |
| --- | --- | --- | --- |
| Control (Median) | -0.28517 | -0.1008 | 0.059982 |
| COVID (Median) | 0.086223 | 0.01579 | 0.032195 |
|  |  |  |  |
| p value Wilcoxon | <0.0001 | 0.02 | 0.9 |

| **At Genus level** | **PC1** | **PC2** | **PC3** |
| --- | --- | --- | --- |
| Control (Median) | -0.34489 | -0.11894 | 0.087134 |
| COVID (Median) | 0.14897 | 0.067733 | 0.012532 |
|  |  |  |  |
| p value Wilcoxon | <0.0001 | 0.01 | 0.2 |

Table S3: PCoA co-ordinate values for PC1, PC2 and PC3 axes among Delta, Omicron infected patients and Healthy controls

| **At Species level** | **PC1** | **PC2** | **PC3** |
| --- | --- | --- | --- |
| Control (Median) | -0.28517 | -0.1008 | 0.059982 |
| Delta (Median) | 0.31428 | 0.27576 | 0.048357 |
| Omicron (Median) | -0.14111 | -0.04701 | 0.0229 |
|  |  |  |  |
| p value Wilcoxon Control vs Delta | <0.001 | 0.02 | 0.3 |
| p value Wilcoxon Control vs Omicron | 0.1 | <0.001 | 0.2 |

| **At Genus level** | **PC1** | **PC2** | **PC3** |
| --- | --- | --- | --- |
| Control (Median) | -0.34489 | -0.11894 | 0.087134 |
| Delta (Median) | 0.39614 | 0.24753 | 0.019119 |
| Omicron (Median) | -0.02425 | -0.02769 | 0.011042 |
|  |  |  |  |
| p value Wilcoxon Control vs Delta | <0.0001 | 0.03 | 0.2 |
| p value Wilcoxon Control vs Omicron | 0.2 | <0.001 | 0.5 |

Table S4: Comparison with other URT microbiome studies on COVID-19

|  |  |  |  |  | **Remarks** | | | | | |
| --- | --- | --- | --- | --- | --- | --- | --- | --- | --- | --- |
| **Serial No.** | **Paper Title** | **Sample Size** | **Sequencing Method** | **Reference** | **Pseudomonas** | **Acinetobacter** | **Streptococcus** | **Prevotella** | **Neisseria** | **Staphylococcus** |
| 1 | Nasopharyngeal microbiome of COVID-19 patients revealed a distinct bacterial profile in deceased and recovered individuals | a. 97patients b. 44controls | 16S rRNA gene (V1-V3) sequencing | Kumar et al., 2020 | High in cases | High in cases | High in controls | High in controls | High in controls | High in cases |
| 2 | Nasopharyngeal Microbiota in SARS-CoV-2 Positive and Negative Patients | a. 9patients b. 10controls | 16S rRNA gene (V3-V4) sequencing | Engen et al., 2021 | High in cases | NA | High in controls | High in controls | NA | High in cases |
| 3 | Acute SARS-CoV-2 infection is associated with an increased abundance of bacterial pathogens, including Pseudomonas aeruginosa in the nose | a. 68 patients b. 45 health care worker control c. 21 outpatients | 16S rRNA gene (V4) sequencing | Rhoades et al., 2021 | High in cases | High in cases | High in controls | NA | High in controls | High in controls |
| 4 | Bacterial microbiota in upper respiratory tract of COVID-19 and influenza patients | a. 24 patients b. 24 controls without COVID-19 | 16S rRNA gene (V4) sequencing | Rattanaburi et al., 2022 | High in cases | NA | High in controls | High in controls | NA | High in cases |
| 5 | Super Dominant Pathobiontic Bacteria in the Nasopharyngeal Microbiota Cause Secondary Bacterial Infection in COVID-19 Patients | a. 51 mild patients b. 20 severe patients | 16S rRNA gene (V3-V4) sequencing | Qin et al., 2022 | High in cases | High in cases | NA | NA | NA | High in cases |
| 6 | SARS-CoV-2 infection and viral load are associated with the upper respiratory tract microbiome | a. 38 patients b. 21 controls | 16S rRNA gene (V4) sequencing | Rosas-Salazar et al., 2021 | NA | NA | NA | High in controls | High in controls | High in controls |
| 7 | Severe COVID-19 is Associated With an Altered Upper Respiratory Tract Microbiome | a. 27 mild patients b. 28 moderate patients c. 15 severe patients d. 13 very severe(ICU)patients e. 20 control | 16S rRNA gene (V4) sequencing | Shilts et al., 2022 | NA | NA | NA | NA | High in controls | High in cases |
| 8 | Our Study | a. 19 omicron b. 24 delta c. 19control | 16S rRNA gene (V3-V4) sequencing | Nath et al., | High in cases | High in cases | High in controls | High in controls | High in controls | High in cases |

a)


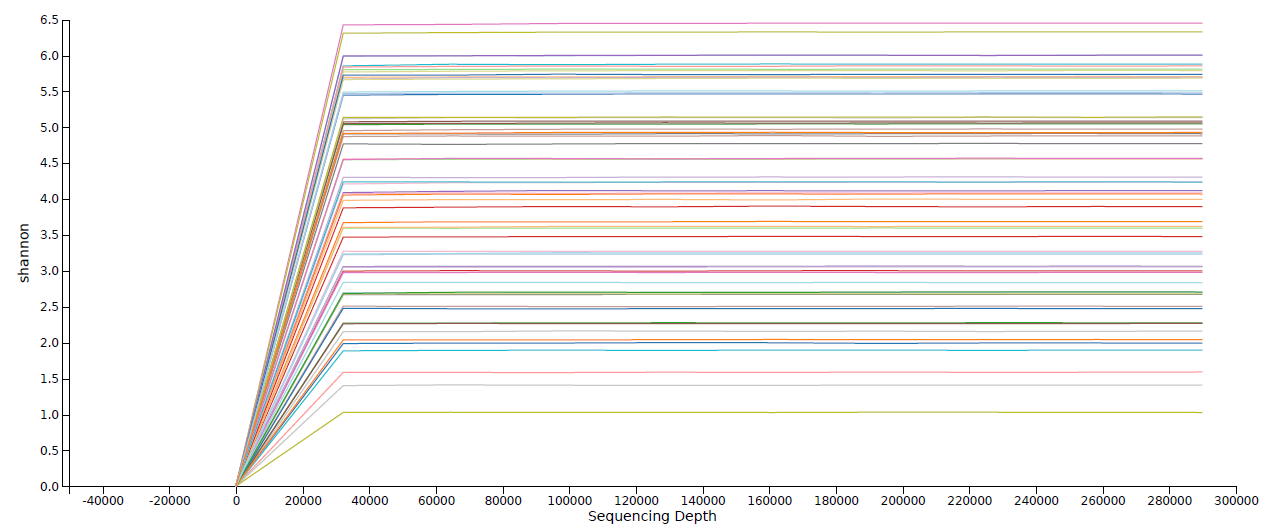


b)


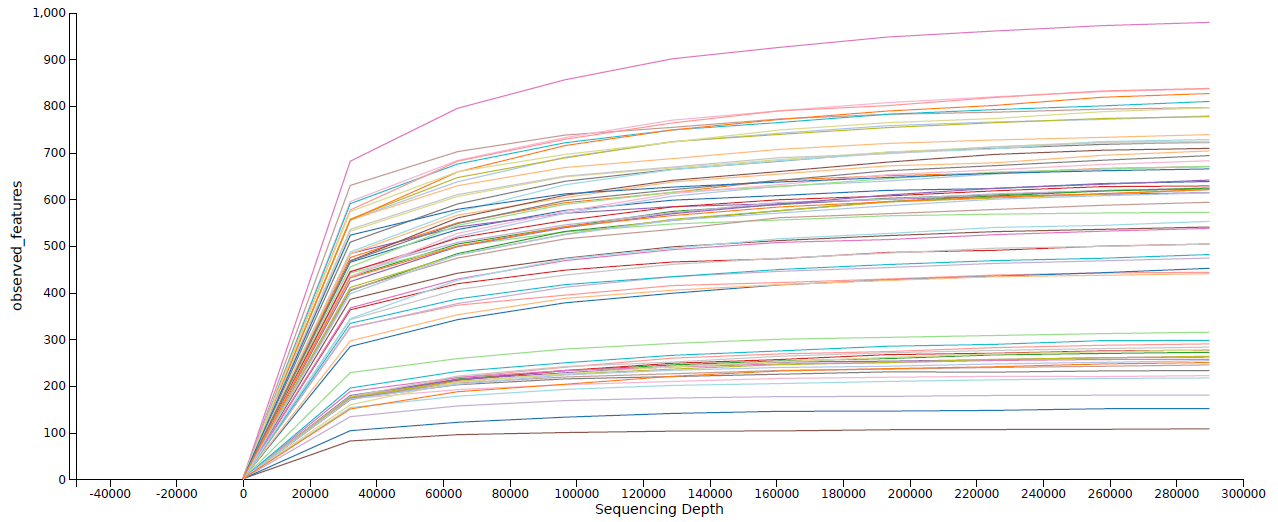


**Figure S1 –Rarefaction curve of alpha diversity indices of microbial communities**. a) – Shannon (species evenness), b) – Observed Features (Amplicon Sequence Variants).

a)

b)

c)

**Figure S2 –Regression plot between viral load (Ct value) and core microbiome composition**. Spearman rank correlation test was performed and shown in the plot, where “rho” denotes correlation coefficient; “p” denotes level of significance.*Streptococcussymci*(a)*,Streptococcus toyakuensis*(b)were significantly positively and *Pseudomonas aeruginosa*(c)was significantly negatively correlated with the Ct Valuerespectively.


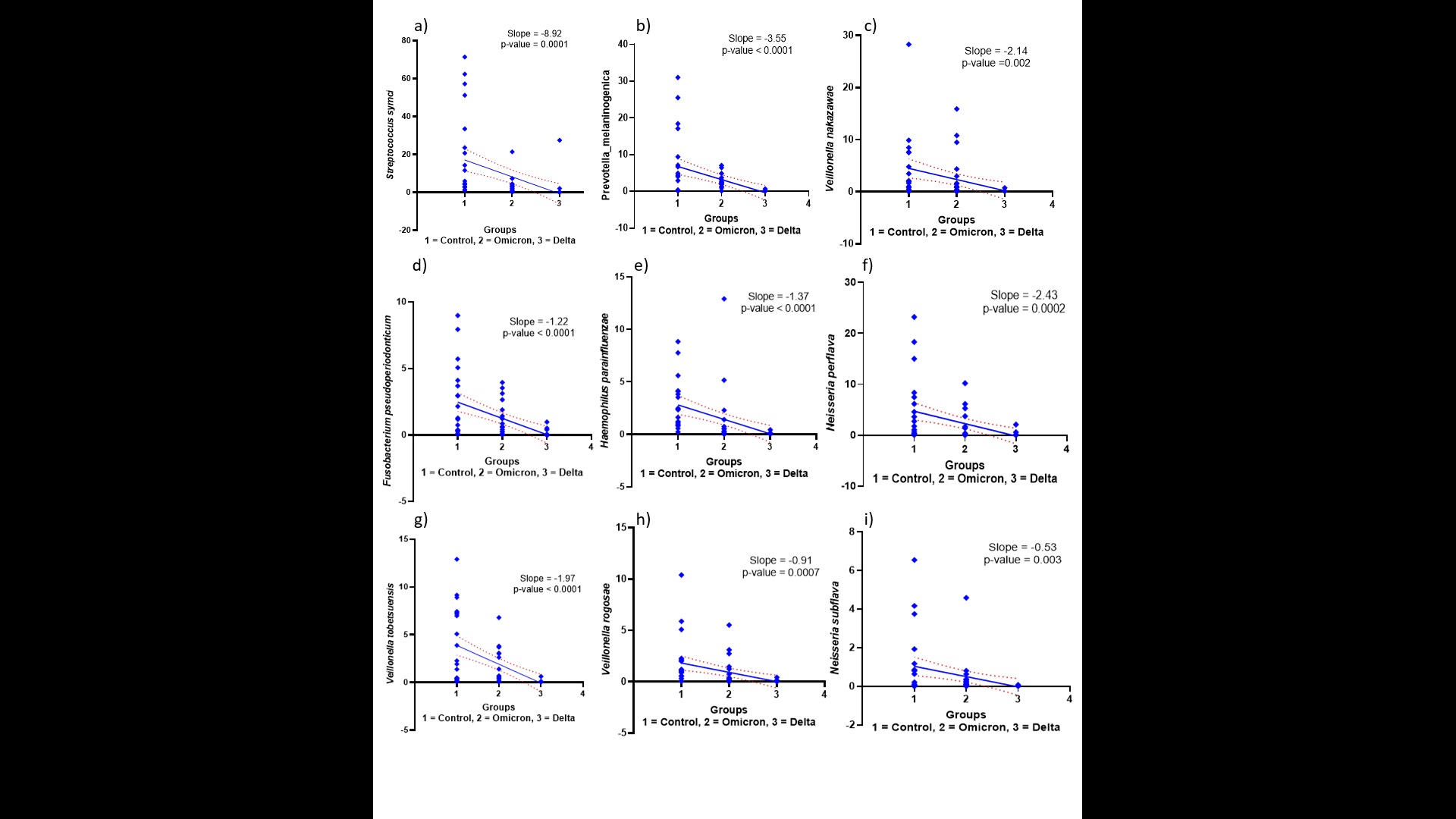


**Figure S3 –Multinomial regression analysis showing trend of nine species**. Regression plot showing 9 species (a – i) having higher to lower abundance from Control>Omicron>Delta based on significantly lower value in slope from zero (slope < 0) by post hoc test for linear trend (p < 0.05). Dependent variable: relative abundance of taxa (Y axis), Independent variable: Groups (1=Control, 2=Omicron, 3=Delta).


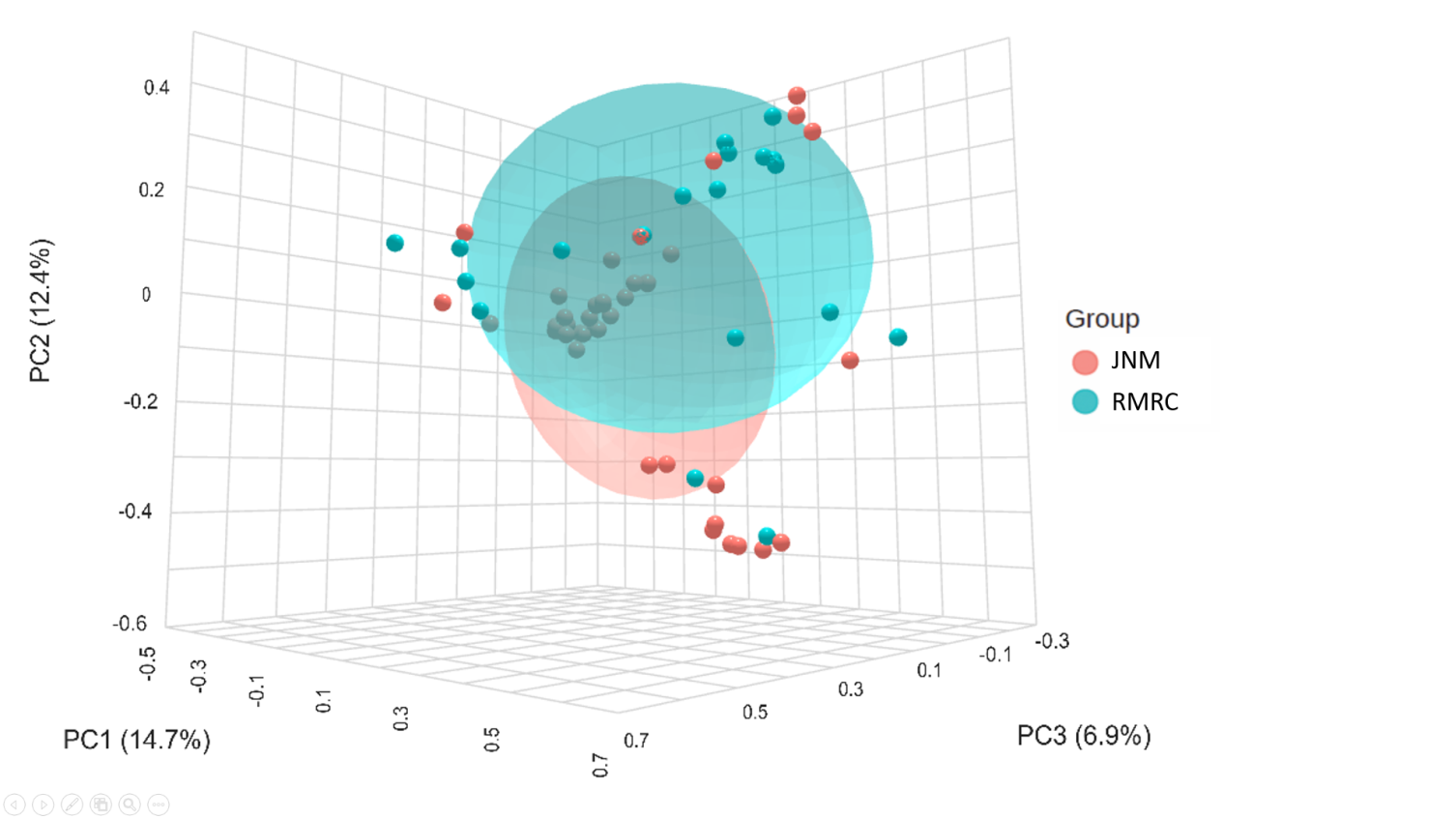


[PERMANOVA] F-value: 1.4748; R-squared: 0.054743; p-value: 0.11

**Figure S4** –**Principal Coordinate Analysis (PCoA) based on Bray-Curtis dissimilarity index showsJNM (Red dots) and RMRC (Green Dots) samples are clustered together.**
